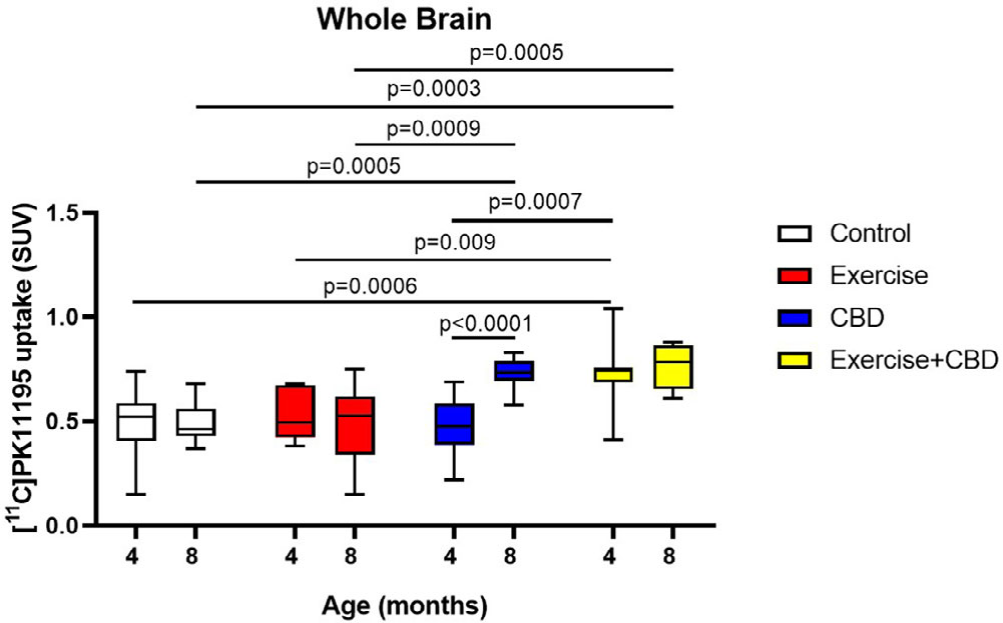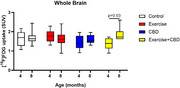# Exploring the synergistic effects of physical exercise and cannabidiol treatment in a mouse model of Down syndrome using positron emission tomography

**DOI:** 10.1002/alz70856_099067

**Published:** 2025-12-25

**Authors:** Chiara Maria Righini, Larissa Estessi de Souza, Jean Marques Brizola, Carlos Alberto Buchpiguel, Lidia Emmanuela Wiazowski Spelta, Daniele de Paula Faria

**Affiliations:** ^1^ University of São Paulo Medical School, São Paulo, São Paulo, Brazil; ^2^ Universidade de São Paulo, São Paulo, SP, Brazil

## Abstract

**Background:**

Down Syndrome (DS) is considered a genetic form of Alzheimer's disease (AD) as it increases the expression of AD‐related genes, leading to precocious neuroinflammation and cell death. Given the increasing longevity of the DS population, it is essential to look for alternatives to delay AD development and improve quality of life. Considering that physical exercise and cannabidiol (CBD) have recognized neuroprotective properties, the aim of this study was to evaluate the effects of these interventions alone and in combination, in a model of DS using positron emission tomography (PET).

**Method:**

Ts65Dn trisomic mice (ethical approval: 1292/2019, 1811/2022, 2065/2024) were divided into four groups: control (no treatment); exercise; CBD; and exercise+CBD. The exercise group was subjected to physical exercise on a treadmill from 2 to 8 months of age, 3 times/week, and the CBD group was treated at 7 months of age for 30 days (20 mg/kg, ip). The exercise+CBD group received both treatments as described above. PET images with [^11^C]PK11195 and [^18^F]FDG were acquired to assess brain metabolism and neuroinflammation at 4 and 8 months of age using a small animal PET scanner. The average standardized uptake value (SUV) was calculated considering the whole brain (WB) and the hippocampus.

**Result:**

At 4 months, exercise+CBD had higher [^11^C]PK11195 uptake in the WB and hippocampus compared to the other groups (control: *p* = 0.0006 in both; exercise: *p* = 0.009 and *p* = 0.005; CBD: *p* = 0.0007 and *p* = 0.0006). At 8 months, CBD and CBD+exercise had higher radiotracer uptake compared to the control (WB: *p* = 0.0005 and *p* = 0.0003, respectively; hippocampus: *p* = 0.0002 and *p* = 0.0003, respectively) and exercise groups (WB: *p* = 0.0009 and *p* = 0.0005, respectively; hippocampus: *p* <0.0001 for both). The CBD group showed increased [^11^C]PK11195 uptake in the WB (*p* <0.0001) and hippocampus (*p* <0.0001) at 8 months of age. Regarding [^18^F]FDG, the exercise+CBD group had an increase in uptake from 4 to 8 months in both regions (WB: *p* = 0.03; hippocampus: *p* = 0.04).

**Conclusion:**

Our data suggest that CBD treatment induced a higher glial response that was not present when CBD was associated with physical exercise. Indeed, this association increased brain metabolism, suggesting a neuroprotective mechanism.